# Comparative Efficacy and Safety of Targeted Therapies for Chronic Thromboembolic Pulmonary Hypertension: A Systematic Review and Network Meta-Analysis

**DOI:** 10.1155/2021/1626971

**Published:** 2021-09-01

**Authors:** Yusi Chen, Fang Li, Jun Luo, Jingyuan Chen, Peng Luo, Jiang Li

**Affiliations:** ^1^Department of Cardiology, The Second Xiangya Hospital of Central South University, No. 139 Middle Renmin Road, Furong District, Changsha, Hunan 410011, China; ^2^Department of Radiology and Imaging, Zhuzhou Central Hospital of Central South University, No. 116 Changjiang South Road, Tianyuan District, Zhuzhou, Hunan 410011, China

## Abstract

**Background:**

There is significant controversy relating to whether chronic thromboembolic pulmonary hypertension (CTEPH) can be treated with pulmonary arterial hypertension- (PAH-) targeted therapies and which therapy is the optimal choice for patients. A large number of randomized controlled trials (RCTs) have compared PAH-targeted therapies with placebo or conventional therapies. In this study, we aimed to compare all of the PAH-targeted medications that are used to treat CTEPH and rank their efficacy by the application of network meta-analysis (NMA).

**Methods:**

We searched PubMed, EMBASE, Web of Science, the Cochrane Central Register, https://clinicaltrials.gov, and who.int/trialsearch/, for relevant RCTs published up to January 2020. In addition to traditional meta-analysis, we also performed NMA in our systematic review, as deployed in a previous protocol (PROSPERO: CRD42020173765).

**Results:**

Our study identified eight eligible RCTs that evaluated seven PAH-targeted therapies in 703 patients with CTEPH. NMA revealed that riociguat was ranked first as the most optimized therapy for ameliorating the 6-minute walk distance with a probability of 80.4%. Bosentan was significantly better than others with regard to reducing brain natriuretic peptide/N-terminal pro-B-type natriuretic peptide with a probability of 84.3%. Sildenafil was identified as the best drug in terms of improving the New York Heart Association/World Health Organization functional class with a probability of 87.3%. Treprostinil and macitentan were more beneficial than other drugs in reducing pulmonary vascular resistance and lowering the incidence of clinical worsening with probabilities of 86.2% and 79.2%, respectively.

**Conclusion:**

Analysis revealed positive advantages for the use of PAH-targeted drugs in patients with CTEPH. Overall, treprostinil and riociguat were superior to all other PAH-targeted medications in most of the outcomes investigated.

## 1. Introduction

Chronic thromboembolic pulmonary hypertension (CTEPH), classified as group 4 pulmonary hypertension (PH) [1] by the World Health Organization (WHO), is a potentially lethal disease. CTEPH is characterized by pulmonary thrombosis and pulmonary vascular remodelling and results in a progressive increase in pulmonary arterial pressure and pulmonary vascular resistance (PVR) with a poor prognosis.

Unlike other forms of PH, CTEPH can be rectified by surgery, at least in theory [2]. For appropriate patients with CTEPH, pulmonary endarterectomy (PEA) is the best treatment option; this procedure involves the removal of chronic thromboembolic clots from the proximal vessel and dissects the endothelium and part of the media. However, not all patients with CTEPH are suitable for this surgery; four issues need to be taken into consideration: (1) an extensive blockage in the distal precapillary is not accessible by surgery; (2) up to 50% of patients develop residual CTEPH after PEA [3]; (3) patients with severe hemodynamic condition need a bridge to the surgical procedure; and (4) this form of surgery carries a notable risk for patients with significant comorbidities. For these patients, the current guidelines recommend the consideration of balloon pulmonary angioplasty (BPA) as an alternative [4, 5]. However, whether patients with CTEPH who are not suitable for PEA can be treated with pulmonary arterial hypertension- (PAH-) targeted medications remains controversial.

The changes in pulmonary vessels and hemodynamics observed in CTEPH are similar to those seen in patients with PAH. Furthermore, the biopsies of small pulmonary arteries from patients with CTEPH have been shown to be comparable to specimens from patients with PAH [6]. These findings suggest that PAH and CTEPH probably share common pathophysiological contexts [7], thus providing reasonable evidence to suggest that patients with CTEPH can take PAH-targeted drugs. Targeted therapies include soluble guanylate cyclase stimulator (sGC), endothelin receptor antagonists (ERAs), phosphodiesterase-5 inhibitors (PDE5i), prostacyclin and its analogs, and prostacyclin-receptor agonists.

Although randomized controlled trials (RCTs) [8–15], and a previous traditional meta-analysis [16], have compared individual drugs to placebo or conventional therapy, evidence regarding the most optimized treatment, and comparing different PAH-specific medications with each other in terms of efficacy and safety, is very limited. The previous meta-analysis only revealed that PAH‐targeted therapies are associated with positive advantages in certain outcomes when compared with placebo for patients with CTEPH [16]. Both the number of RCTs included in the previous meta-analysis and the number of outcomes described were smaller than in our current analysis featuring network meta-analysis (NMA). Furthermore, pairwise meta-analyses are limited by estimates between two interventions when compared directly with each other; this form of analysis is also unable to compare all of the available interventions. These issues can be addressed by NMA [17], a tool that can allow for the simultaneous evaluation of multiple interventions and provide valuable indirect comparisons in the absence of direct comparisons. The most significant advantage of NMA is its ability to aggregate different interventions for the treatment of similar diseases and then compare these in a quantitative manner that will allow detailed statistical analysis [18]. NMA is also able to rank different interventions based on their therapeutic effects and identify the probability of optimal interventions.

In the present study, we considered all relevant RCTs that aimed to evaluate the efficacy and safety of PAH-targeted drugs in patients with CTEPH. We then used NMA to rank the drug treatments based on evidence described in the literature. We intend to provide guidelines to facilitate the recommendation of specific drug treatments for patients with CTEPH.

## 2. Materials and Methods

This study, including its inclusion and exclusion criteria, literature sources and searches, data extraction, quality assessment and risk of bias assessment, and data synthesis and analysis, was conducted under the Cochrane criteria [19] and the PRISMA guidelines [20, 21]. Items for the systematic review and NMA were identified based on an a priori protocol (PROSPERO: CRD42020173765) [22].

### 2.1. Inclusion and Exclusion Criteria

We included RCTs in our analysis if they met the following criteria: (1) study objects: patients older than 18 years with symptomatic CTEPH confirmed by right heart catheterization, irrespective of gender, according to the New York Heart Association (NYHA)/WHO functional class (NYHA/WHO FC); (2) interventions: the use of at least one prostacyclin and its analogs (epoprostenol, iloprost, treprostinil, and selexipag), ERAs (bosentan, ambrisentan, and macitentan), PDE5i (sildenafil, tadalafil, and vardenafil), and sGC (riociguat), regardless of drug dosage forms; (3) study type: single or double-blinded RCTs published between January 1980 and January 2020, with no limitation to language; and (4) outcomes: trials reporting at least one efficacy outcome (e.g., the 6-minute walk distance (6MWD), brain natriuretic peptide (BNP)/N-terminal pro-B-type natriuretic peptide (NT-proBNP), improvement in the NYHA/WHO FC, or PVR), or safety outcome (clinical worsening). Patients were excluded if they had other forms of PH. We also excluded publications if they featured duplicated data; in these cases, we only retained the most recent papers or the most comprehensive papers.

### 2.2. Literature Sources and Searches

**Figure 1:**
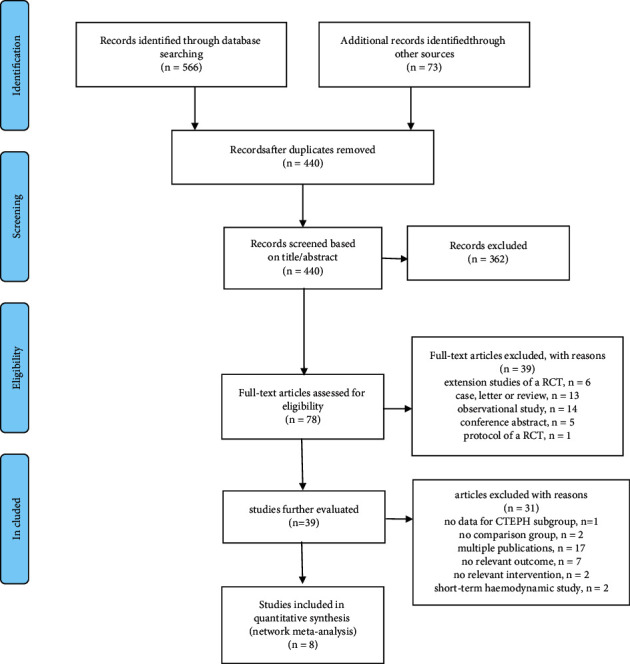
Flow chart of study selection.

We searched multiple databases for RCTs related to the pharmacological therapy of patients with CTEPH, including PubMed, EMBASE, Web of Science, and the Cochrane Central Register of Controlled Trials, for publications dated on or before 16^th^ January 2020. We also searched the US National Institutes of Health Ongoing Trials Register ClinicalTrials.gov (https://www.clinicaltrials.gov) and the World Health Organization International Clinical Trials Registry Platform (https://apps.who.int/trialsearch/). Medical subject headings and text words related to patients with CTEPH and all PAH-specific drugs were used to develop a specific and consistent search strategy, thus providing maximum sensitivity for the detection of PAH-specific therapeutic trials in CTEPH. The authors independently scrutinized the titles and abstracts as stated by the inclusion criteria. We then acquired the full texts for all titles that appeared to meet the inclusion criteria or for which there was any uncertainty. The authors then reviewed the full text of each paper and decided whether these met the inclusion criteria. The rationale for excluding trials is depicted in Figure 1. We resolved any discrepancies by discussion; or, if necessary, a third author was needed to seek consensus and an inter-rater agreement would be calculated. At each stage of the review, the reviewers were blinded to the journal titles, the study authors, and the name of the institutions involved; literature screening was performed twice to reduce the possibility of excluding relevant reports.

### 2.3. Data Extraction and Evaluating the Risk of Bias

Two authors extracted data from the included studies in an independent manner using a standardized data abstraction form. The extracted data included the following: (1) methods: study design, duration, study setting, date of the study, and allocation concealment; (2) participants: number, mean age and age range, gender, inclusion and exclusion criteria, differences in baseline characteristics, and the number of dropouts; (3) intervention: type of PAH-targeted drugs, dose, mode of administration, control drug, and duration of treatment; (4) outcomes: primary outcomes: 6MWD, improvement in the NYHA/WHO FC (defined by an improvement in the FC versus baseline by at least one class), BNP/NT-proBNP; and (5) secondary outcomes: PVR, and clinical worsening. We used the Cochrane Collaboration tool [23, 24] to analyze the potential risk of bias for each study.

### 2.4. Data Analysis and Synthesis

#### 2.4.1. Direct Meta-Analysis

Pairwise meta-analysis was used to perform direct comparisons where trials were considered to be clinically homogenous. The fixed effect, inverse-variance/Mantel–Haenszel model was, respectively, used for continuous data and binary variables when tests of heterogeneity were not significant. If statistical heterogeneity was observed (*I*^2^ ≥ 30%), then the random effect model was implemented to improve the accuracy of research and explore the possible sources of heterogeneity using prespecified subgroup analyses. *p* < 0.05 for the overall estimate is significant.

#### 2.4.2. Network Meta-Analysis

NMA was conducted by the multivariate meta-analysis command in Frequentist. First, we generated a schematic diagram of the network relationship involving several specific drugs [25]. Thus, our NMA involved the consistency model and multivariate fixed-effects analysis. We also ranked different targeted drugs based on primary and secondary outcomes by applying the surface under the cumulative ranking (SUCRA) tool and predicted the ranking probability. Higher estimated probabilities for SUCRA that were close to 1 indicated superiority over other therapies whereas lower values close to 0 indicated inferiority. Therefore, the SUCRA values denote the probability that a treatment is the most ideal, with higher values indicating greater odds in terms of benefit. Further details of this analysis are given in Supplementary Material 1.

## 3. Results

### 3.1. Literature Retrieval and Network Chart Construction

**Figure 2:**
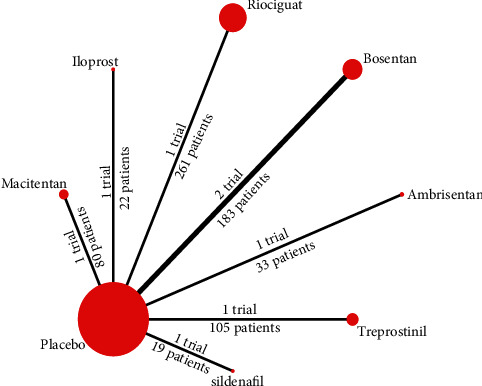
Network structure of included trials for CTEPH. The area of nodes is proportional to the number of patients for each intervention, and the thickness of lines to the number of direct comparisons.

In total, we retrieved 566 articles from databases and 73 registrations. Of these, eight RCTs were ultimately identified for inclusion in our systematic review and NMA. Figure 1 describes the process used to select articles. The identified RCTs involved seven different targeted treatments (iloprost, bosentan, sildenafil, riociguat, macitentan, ambrisentan, and treprostinil) and 703 patients with CTEPH. Of the eight RCTs, none involved head-to-head analysis; only parallel trials between 1 intervention and placebo were evident. The network plot shown in Figure 2 illustrates the direct comparisons across outcomes. Specific outcome network plots for 6MWD, BNP/NT-proBNP, NYHA/WHO FC improvement, PVR, and clinical worsening are presented in Supplementary Figures 1A–1E.

### 3.2. Characteristics of Trials

We identified eight RCTs for inclusion in our final analysis. These were published between October 2005 and March 2019 and included 703 patients (range: 19–261 participants); of these, 394 were assigned to a treatment group and 309 were assigned to a control group. The median period of study follow-up was 16 weeks (ranging from hospital discharge to 24 weeks). The median age of the subjects across all trials was 59 years (range: 36.8–79.2 years) and 62% were female (range: 30%–78%).  Table 1 provides further details relating to the core characteristics of individual trials [8–15].

### 3.3. Risk of Bias Assessment

Supplementary Figure 2 presents the risk of bias assessment relating to the quality of each included trial. Although all trials referred to randomization, only four RCTs (50%) described how they generated random sequences. None of the studies reported allocation concealment. All of the RCTs were double-blinded, except for Reesink (2010); this was a single-blinded trial in which patients knew whether they received bosentan or not, but evaluators were blinded. Overall, the selected studies had a low risk of bias but did not provide information about other forms of bias.

### 3.4. Heterogeneity and Consistency

The calculated *I*^2^ statistics for the 6MWD, BNP/NT-proBNP, NYHA/WHO FC improvement, PVR, and clinical worsening were 24.48%, 0.00%, 27.41%, 15.37%, and −295.54%, respectively. These values suggest that the extent of heterogeneity was too small to limit quantitative pooling. Considering that our network structure was a star-shaped diagram (Figure 2), that is, our NMA only contains indirect evidence to merge, there was no source of inconsistency between indirect evidence and direct evidence (face-to-face RCTs) and, therefore, the evaluation of consistency was meaningless.

### 3.5. Outcomes

The main outcomes of interest for this analysis were 6MWD, BNP/NT-proBNP, and NYHA/WHO FC improvement. Additional outcomes were PVR and clinical worsening. The endpoints used showed variation when considered across the different studies (Supplementary Table 1).

#### 3.5.1. Pairwise Meta-Analysis

We carried out direct comparisons between PAH-targeted therapies and placebos in terms of specific outcomes to determine whether targeted drugs were associated with definitive advantage or harm when used to treat CTEPH patients. The Forest plot shown in Supplementary Figure 3A shows that riociguat, macitentan, and ambrisentan significantly improved the weighted mean for 6MWD in patients with CTEPH when compared to the placebo (riociguat: weighted mean difference (WMD) = 45.00 meters, 95% confidence interval (CI) = 24.29–65.71 meters; macitentan: WMD = 34.00 meters, 95% CI = 3.50–64.50 meters; ambrisentan: WMD = 41.60 meters, 95% CI = 17.01–66.19 meters). When we considered the change in BNP/NT-proBNP from baseline to the end of the studies, we found that none of the treatments showed a significant effect, except for bosentan, as shown in Supplementary Figure 3B (bosentan: standardized mean difference (SMD) = −0.51 pg/ml, 95% CI = −0.84–−0.19 pg/ml). Determining the odds ratios for the improvement in NYHA/WHO FC showed that the amelioration of FC was correlated with two PAH-targeted drugs when compared to placebo, as shown in Supplementary Figure 3C (riociguat: odds ratio (OR) = 2.80, 95%CI = 1.43–5.46; treprostinil: OR = 4.88, 95% CI = 1.94–4.68). With respect to cardiopulmonary hemodynamics, we found that iloprost, bosentan, riociguat, and ambrisentan could all significantly reduce PVR, as shown in Supplementary Figure 3D (iloprost: WMD = -167.00 dynes·sec·cm^−5^, 95% CI = −223.65–−110.35 dynes·sec·cm^−5^; bosentan: WMD = −176.00 dynes·sec·cm^−5^, 95% CI = −257.93–−94.07 dynes·sec·cm^−5^; riociguat: WMD = −249.00 dynes·sec·cm^−5^, 95% CI = −318.21–−179.79 dynes·sec·cm^−5^; ambrisentan: WMD = -287.20 dynes·sec·cm^−5^, 95% CI = −410.07–−164.33 dynes·sec·cm^−5^). None of these specific therapies were associated with significant advantages over placebos with regard to clinical worsening, as shown in Supplementary Figure 3E.

#### 3.5.2. Network Meta-Analysis Results

**Figure 3:**
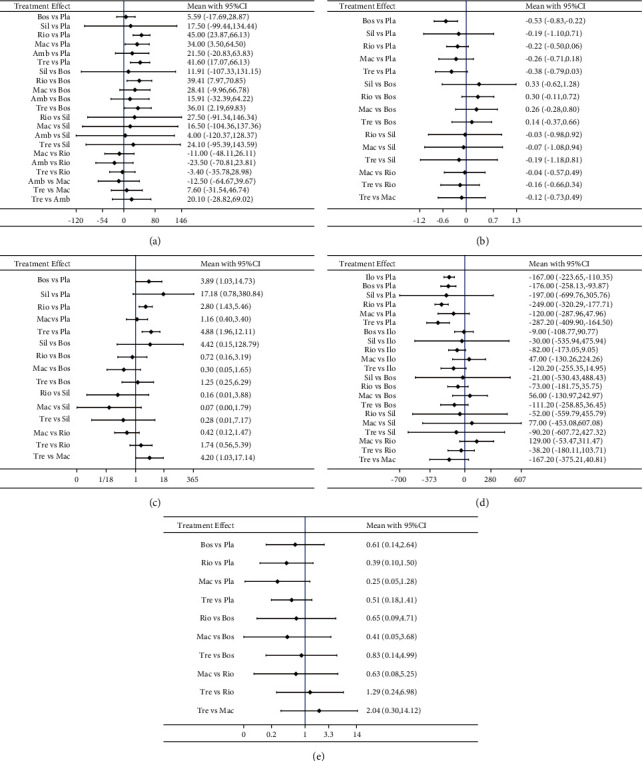
(a–e) Predictive interval plot for all endpoints. The predictive interval plot denotes a forest plot of the estimated summary effects from indirect comparisons along with their 95% CI. (a) 6MWD; (b) BNP/NT-proBNP; (c) NYHA/WHO FC; (d) PVR; (e) clinical worsening. Ilo: iloprost, Bos: bosentan, Sil: sildenafil, Rio: riociguat, Mac: macitentan, Amb: ambrisentan, and Tre: treprostinil.

Changes in the 6MWD, a common indicator used to measure the efficacy of treatments for CTEPH, were described by seven RCTs with six types of targeted drugs. The predictive interval plot shown in Figure 3(a) indicates that only three interventions exhibited a statistically significant difference when compared with placebos: riociguat (WMD = 45.00 meters, 95% CI = 23.87–66.13 meters), treprostinil (WMD = 41.60 meters, 95% CI = 17.07–66.13 meters), and macitentan (WMD = 34.00 meters, 95% CI = 3.50–64.50 meters). The SUCRA rankings shown in Table 2 provide further evidence that riociguat (80.4%), treprostinil (74.6%), and macitentan (64.0%) had significant effects. Riociguat (WMD = 39.41 meters, 95% CI = 7.97–70.85 meters) and treprostinil (WMD = 36.01 meters, 95% CI = 2.19–69.83 meters) were shown to exhibit a significant advantage over bosentan, as shown in Table 3.

Data relating to BNP/NT-proBNP were reported by seven RCTs and six types of targeted therapies; however, ambrisentan was not pooled in the present study due to a lack of specific values; in some cases, data were given as a percentage of baseline. Bosentan (SMD = −0.53 pg/ml, 95% CI = −0.83–−0.22 pg/ml) was the only drug that significantly outperformed the placebo, as shown in Figure 3(b) and Table 3. The SUCRA values shown in Table 2 show that bosentan (84.3%), treprostinil (65.9%), and macitentan (49.8%) were the top-ranked drugs for reducing BNP/NT-proBNP.

Improvements in the NYHA/WHO FC were reported by five RCTs and involved five comparisons. As shown in the predictive interval plot (Figure 3(c)) and Table 3, treprostinil (OR = 4.88, 95% CI = 1.96–12.11) and riociguat (OR = 2.80, 95% CI = 1.43–5.46) performed better than the control group. Moreover, treprostinil (OR = 4.20, 95% CI = 1.03–17.14) also demonstrated an obvious benefit over macitentan. However, when considering the SUCRA results presented in Table 2, it was clear that sildenafil (87.3%) exhibited the highest probability to provide optimal improvement in the NYHA/WHO FC, followed by treprostinil (72.5%) and bosentan (63.2%).

PVR was measured by six RCTs involving six types of specific drugs. The predictive interval plot (Figure 3(d)) and Table 3 show that treprostinil (WMD = −287.20 dynes·sec·cm^−5^, 95% CI = -409.90–−164.50 dynes·sec·cm^−5^), riociguat (WMD = -249.00 dynes·sec·cm^−5^, 95% CI = −320.29–−177.71 dynes·sec·cm^−5^), bosentan (WMD = −176.00 dynes·sec·cm^−5^, 95% CI = -258.13–−93.87 dynes·sec·cm^−5^), and iloprost (WMD = -167.00 dynes·sec·cm^−5^, 95% CI = −223.65–−110.35 dynes·sec·cm^−5^) were all significantly better than the placebo in terms of specific effects on PVR. Treprostinil (86.2%) was ranked as the most effective drug for reducing the PVR, closely followed by riociguat (77.4%) and sildenafil (54.9%).

Clinical worsening was described by four RCTs involving four types of targeted drugs. There was no statistically significant difference in terms of the effects of treatment on reducing clinical worsening, as shown in Figure 3(e) and Table 3. However, SUCRA values showed that macitentan (79.2%) was likely to exert the best effect, followed by riociguat (63.3%), treprostinil (52.6%), and bosentan (42.8%).

## 4. Discussion

The use of PAH‐targeted drugs for the treatment of CTEPH has received considerable levels of attention over the last decade. With more and more RCTs of PAH-targeted medications for the treatment of CTEPH, our study aims to solve whether it is effective and safe to use all kinds of active agents for patients with CTEPH based on RCTs and more importantly which medication is more preferred for patients.

In this systematic review, we used NMA to evaluate the efficacy and safety of seven targeted drugs (iloprost, bosentan, sildenafil, riociguat, macitentan, ambrisentan, and treprostinil); our analyses determined ranking probabilities for each type of drug in terms of five different aspects. Our analyses identified some key discoveries that are more comprehensive than described previously. First, we found that treprostinil performed in an outstanding manner in all of the outcomes investigated. Secondly, riociguat was shown to be the best performing drug in terms of improving the 6MWD; this drug also performed well in terms of reducing PCR and clinical worsening. Bosentan performed significantly better than the other drugs in terms of lowering BNP/NT-proBNP. With regard to improving the NYHA/WHO FC, reducing PVR, and reducing the incidence of clinical worsening, we found that sildenafil, treprostinil, and macitentan were the most beneficial drugs.

Although our study demonstrated that other PAH-targeted medications provided benefit to patients with CTEPH, we found that riociguat was the only drug that is approved for patients with CTEPH [26]. Riociguat ranked first in terms of improving 6MWD and was also ranked second in terms of the probability to reduce PVR; however, the other medications were comparable in certain aspects. In clinical practice, many other targeted drugs are also used to treat patients with CTEPH. One previous study reported the significant recovery of a patient with inoperable and progressive CTEPH who was treated with riociguat and inhaled treprostinil [27]. An open-label study observed significant improvements in the WHO FC, 6MWD, PVR, BNP, and 5-year survival rate, in patients with severe and inoperable CTEPH [28]. In our study, we ranked changes in different outcomes and found that treprostinil, a prostacyclin analog, performed well in every aspect. Treprostinil had the highest probability with regard to reducing PVR, the second-highest probability in terms of improving the 6MWD, BNP/NT-proBNP, and NYHA/WHO FC, and the third-highest probability in terms of reducing the incidence of clinical worsening. Treprostinil may, therefore, represent a potential PAH-targeted medication with which to treat CTEPH. Prostacyclin and its analogs are powerful vasodilators with established antithrombotic, antiproliferative, and anti-inflammatory properties [29, 30]. Researchers have also investigated other types of prostacyclin analogs, such as epoprostenol and beraprost, in patients with CTEPH; these studies have identified certain benefits [31–33]. However, there is a notable absence of RCTs and the iloprost data are incomplete. Further investigation of these agents may be warranted.

With regard to ERAs, we found that bosentan was associated with the highest probability for lowering BNP/NT-proBNP; this was the only drug that showed significant improvement in this parameter. However, retrospective research has identified that the long-term efficacy of bosentan failed to remain statistically significant in inoperable CTEPH patients [34]. Our results are based on RCTs and are not completely consistent with other published research. This is probably because the condition of patients with CTEPH in the real world is not always the same as the design of the RCTs; for example, sicker patients or CTEPH sub-phenotypes may have more complicated physiology, thus generating bias. We found that macitentan exhibited the highest probability for reducing the incidence of clinical worsening and the third-highest probability for improving 6MWD and BNP/NT-proBNP. Because macitentan outperformed other ERAs, it follows that this drug has significant potential for the treatment of patients with CTEPH.

PDE-5i has been shown to prohibit platelet activation and pulmonary vascular remodelling. Therefore, PDE-5i may also be of benefit for patients with CTEPH, at least in theory; however, our data suggest that the efficacy of PDE-5i has yet been fully determined. In our analysis, sildenafil was ranked first in terms of improving the NYHA/WHO FC; this was followed by treprostinil. Of note, the RCT involving sildenafil only featured a small sample size; consequently, there was a lack of statistical significance when compared against the placebo. Therefore, sildenafil may not actually be superior to treprostinil in terms of ameliorating the NYHA/WHO FC; it is possible that the observed improvement in the NYHA/WHO FC was mainly driven by data arising from the small sample size.

Our study, which indirectly compared the specific effects of different drugs by network meta-analysis, suggests that riociguat is effective for the treatment of CTEPH, but also that other targeted drugs are more superior. This may have implications for clinicians and patients in terms of selecting targeted pulmonary vasodilators to improve the specific parameters that they wish to improve.

Our study has some potential limitations that need to be considered. First, one RCT relating to selexipag and published in September 2020 [35] was not included in our analysis due to our deadline for literature retrieval (January 2020). Second, our analysis was restricted by the small number of RCTs included for each drug. Third, it is important to mention that one study of CTEPH [15] used low-dose subcutaneous treprostinil (approximately 3 ng/kg per min) as a control to allow complete double-blinding for the drug that causes local infusion site reactions and it did little work on patients [36], acting as a placebo. Therefore, we treated data arising from this as a placebo when analyzing our data. This may have led to an underestimation of the efficacy of treprostinil. Fourth, SUCRA values suggested that macitentan might have the highest probability for reducing the incidence of clinical worsening, followed by riociguat. However, this conclusion was significantly limited by the definition of clinical worsening. This term has been defined differently across trials, including combinations of a reduction in the 6MWD of more than 20% from baseline, hospitalization, mortality, and lung transplantation. Fifth, our study is a reflection on the RCTs that have been selected. Whether different rankings for different drugs make biological or clinical sense needs to be considered further. Finally, we need to acknowledge that differences in RCT design, inclusion criteria (CTEPH disease severity and sub-phenotypes), the description of outcome measures, and study size may have affected our results. To minimize these issues, we adopted strict inclusion criteria.

## 5. Conclusions

In summary, we analyzed seven selected PAH-targeted medications for the treatment of CTEPH and found that riociguat and treprostinil were superior to the other five drugs in terms of efficacy and safety. In future studies, more attention should be paid to determining the specific efficacy of different types of PAH-targeted drugs, doses, and modes of administration, in patients with CTEPH.

## Figures and Tables

**Table 1:** Characteristics of included randomized controlled trials comparing specific drugs for treatment of CTEPH.

Author	Year	Trial acronym	Duration	Therapy (sample size)	Age (year)	Female	Male	Dose	Multicenter	Blind
Kramm	2005	—	Hospital discharge	Iloprost (*n* = 11) Placebo (*n* = 11)	54 ± 17 56 ± 13	36% 36%	64% 64%	25 ug	Single	Double
Jais X	2008	BENEFIT	16 weeks	Bosentan (*n* = 77) Placebo (*n* = 80)	63 ± 12.9 63.1 ± 10.3	71% 59%	29% 41%	125 mg bid	Multi	Double
Suntharalingam	2008	—	12 weeks	Sildenafil (*n* = 9) Placebo (*n* = 10)	49.9 ± 13.1 60.0 ± 14.4	78% 30%	22% 70%	40 mg tid	Single	Double
Reesink	2010	—	16 weeks	Bosentan (*n* = 14) Placebo (*n* = 12)	67 ± 8 64 ± 10	71% 66%	29% 34%	125 mg bid	Single	Single
Ghofrani	2013	CHEST-1	16 weeks	Riociguat (*n* = 173) Placebo (*n* = 88)	59 ± 14 59 ± 13	68% 61%	32% 39%	2.5 mg tid	Multi	Double
Ghofrani	2018	MERIT-1	24 weeks	Macitentan (*n* = 40) Placebo (*n* = 40)	58.2 ± 14 56.9 ± 13.9	65% 63%	35% 38%	10 mg qd	Multi	Double
Escribano-Subias P	2019	AMBER 1	16 weeks	Ambrisentan (*n* = 17) Placebo (*n* = 16)	61.2 ± 13.4 59.8 ± 9	47% 63%	53% 37%	5 mg qd	Multi	Double
Sadushi-Kolici R	2019	CTREPH	24 weeks	Treprostinil (*n* = 53) Low-dose Trepro-stinil (*n* = 52)	68 ± 11.2 61 ± 14.6	36% 58%	64% 42%	30 ng/kg/min 3 ng/kg/min	Multi	Double

**Table 2:** Ranking of PAH-targeted drugs for CTEPH assessed by estimated and predictive probabilities using SUCRA values.

Intervention	SUCRA				
	6MWD (%)	BNP (%)	NYHA/WHO FC improvement (%)	PVR (%)	Clinical worsening (%)
Placebo	14.7	11.2	9.0	5.0	12.1
Bosentan	24.2	84.3	63.2	48.4	42.8
Sildenafil	45.6	43.4	87.3	54.9	—
Riociguat	80.4	45.4	50.7	77.4	63.3
Macitentan	64.0	49.8	17.3	33.9	79.2
Ambrisentan	46.4	—	—	—	—
Treprostinil	74.6	65.9	72.5	86.2	52.6
Iloprost	—	—	—	44.2	—

Higher estimated probabilities of SUCRA close to 100% indicate superiority over other therapies, whereas lower values close to 0% indicate inferiority.

**Table 3:** Results of the efficacy and safety of the PAH-targeted drugs according to the network meta-analysis.

6MWD (meters)	Riociguat						
	3.40 (−28.98, 35.78)	Treprostinil					
	11.00 (−26.11, 48.11)	7.60 (−31.54, 46.74)	Macitentan				
	23.50 (−23.81, 70.81)	20.10 (−28.82, 69.02)	12.50 (−39.67, 64.67)	Ambrisentan			
	27.50 (−91.34, 146.34)	24.10 (−95.39, 143.59)	16.50 (−104.36, 137.36)	4.00 (−120.37, 128.37)	Sildenafil		
	**39.41 (7.97, 70.85)**	**36.01 (2.19, 69.83)**	28.41 (−9.96, 66.78)	15.91 (−32.39, 64.22)	11.91 (−107.33, 131.15)	Bosentan	
	**45.00 (23.87, 66.13)**	**41.60 (17.07, 66.13)**	**34.00 (3.50, 64.50)**	21.50 (−20.83, 63.83)	17.50 (−99.44, 134.44)	5.59 (−17.69, 28.87)	Placebo

NYHA/WHO FC	Sildenafil						
	3.52 (0.14, 89.04)	Treprostinil					
	4.42 (0.15, 128.79)	1.25 (0.25, 6.29)	Bosentan				
	6.14 (0.26, 146.24)	1.74 (0.56, 5.39)	1.39 (0.31, 6.17)	Riociguat			
	14.80 (0.56, 392.81)	**4.20 (1.03, 17.14)**	3.35 (0.61, 18.51)	2.41 (0.68, 8.53)	Macitentan		
	17.18 (0.78, 380.84)	**4.88 (1.96, 12.11)**	**3.89 (1.03, 14.73)**	**2.80 (1.43, 5.46)**	1.16 (0.40, 3.40)	Placebo	

BNP/NT-pro BNP (pg/ml)	Sildenafil						
	0.19 (−0.81, 1.18)	Treprostinil					
	0.33 (−0.62, 1.28)	0.14 (−0.37, 0.66)	Bosentan				
	0.03 (−0.92, 0.98)	−0.16 (−0.66, 0.34)	−0.30 (−0.72, 0.11)	Riociguat			
	0.07 (−0.94, 1.08)	−0.12 (−0.73, 0.49)	−0.26 (−0.80, 0.28)	0.04 (−0.49, 0.57)	Macitentan		
	−0.19 (−1.10, 0.71)	−0.38 (−0.79, 0.03)	**−0.53 (−0.83, −0.22)**	−0.22 (−0.50, 0.06)	−0.26 (−0.71, 0.18)	Placebo	

PVR (dynes/sec/cm−5)	Treprostinil						
	−38.20 (−180.11, 103.71)	Riociguat					
	−90.20 (−607.72, 427.32)	−52.00 (−559.79, 455.79)	Sildenafil				
	−111.20 (−258.85, 36.45)	−73.00 (−181.75, 35.75)	−21.00 (−530.43, 488.43)	Bosentan			
	−120.20 (−255.35, 14.95)	−82.00 (−173.05, 9.05)	−30.00 (−535.94, 475.94)	−9.00 (−108.77, 90.77)	Iloprost		
	−167.20 (−375.21, 40.81)	−129.00 (−311.47, 53.47)	−77.00 (−607.08, 453.08)	−56.00 (−242.97, 130.97)	−47.00 (−224.26, 130.26)	Macitentan	
	**−287.20 (−409.90, −164.50)**	**−249.00 (−320.29, −177.71)**	−197.00 (−699.76, 305.76)	**−176.00 (−258.13, −93.87)**	**−167.00 (−223.65, −110.35)**	−120.00 (−287.96, 47.96)	Placebo

Clinical worsening	Treprostinil						
	1.29 (0.24, 6.98)	Riociguat					
			Sildenafil				
	0.83 (0.14, 4.99)	0.65 (0.09, 4.71)		Bosentan			
					Iloprost		
	2.04 (0.30, 14.12)	1.58 (0.19, 13.16)		2.45 (0.27, 22.11)		Macitentan	
	0.51 (0.18, 1.41)	0.39 (0.10, 1.50)		0.61 (0.14, 2.64)		0.25 (0.05, 1.28)	Placebo

Comparisons should be read from the left (active agent) to the right (comparator agent or placebo). Change of 6MWD, with WMD > 0 indicating higher improvement. The change in BNP/NT-proBNP, with SMD < 0 supporting the intervention. NYHA/WHO FC improvement is defined as an increase in NYHA/WHO FC by at least one level, with OR > 1 favouring effective treatment. Clinical worsening, with OR < 1 corresponding to the active agent. PVR change, with WMD < 0 denoting higher amelioration. Bold numbers are statistically significant. Numbers in parentheses indicate 95% CI.

## Abbreviations

WHO: World Health Organization
CTEPH: Chronic thromboembolic pulmonary hypertension
PAH: Pulmonary arterial hypertension
NMA: Network meta-analysis
6MWD: 6-minute walk distance
PVR: Pulmonary vascular resistance
PH: Pulmonary hypertension
PAP: Mean pulmonary artery pressure
PEA: Pulmonary endarterectomy
BPA: Balloon pulmonary angioplasty
GC: Soluble guanylate cyclase stimulator
ERA: Endothelin receptor antagonists
PDE5i: Phosphodiesterase-5 inhibitors
GMP: Cyclic guanosine monophosphate
RCTs: Randomized controlled trials
FC: Functional class
NYHA: New York Heart Association
BNP: Brain natriuretic peptide
NT-proBNP: N-terminal pro-B-type natriuretic peptide
WMD: Weighted mean difference
SMD: Standardized mean difference
OR: Odds ratio
CI: Confidence interval
SUCRA: Surface under the cumulative ranking.

## Supplementary Materials

Supplementary Materials *Supplementary Material 1*. Details about data analysis. *Supplementary Figure 1 A–E*. Network plot for all outcomes. *Supplementary Figure 2.* Risk of bias summary. *Supplementary Figure 3 A–E*. Pairwise meta-analysis for 6MWD, BNP/NT-proBNP, NYHA/WHO FC improvement, PVR, and clinical worsening. *Supplementary Table 1.* Outcome measures are being used in each included RCT.
