# Supplementary material for: Comparative Efficacy and Safety of Targeted Therapies for Chronic Thromboembolic Pulmonary Hypertension: A Systematic Review and Network Meta-Analysis
Source: Can Respir J. 2021 Sep 1;2021:1626971. doi: 10.1155/2021/1626971 (PMC8426079; doi:10.1155/2021/1626971)
Supplement: Supplementary Materials — Supplementary Material 1. Details about data analysis. Supplementary Figure 1 A–E. Network plot for all outcomes. Supplementary Figure 2. Risk of bias summary. Supplementary Figure 3 A–E. Pairwise meta-analysis for 6MWD, BNP/NT-proBNP, NYHA/WHO FC improvement, PVR, and clinical worsening. Supplementary Table 1. Outcome measures are being used in each included RCT. [file 1626971.f1.zip › 1626971.f1/Supplementary Figure 2 (1).pdf]

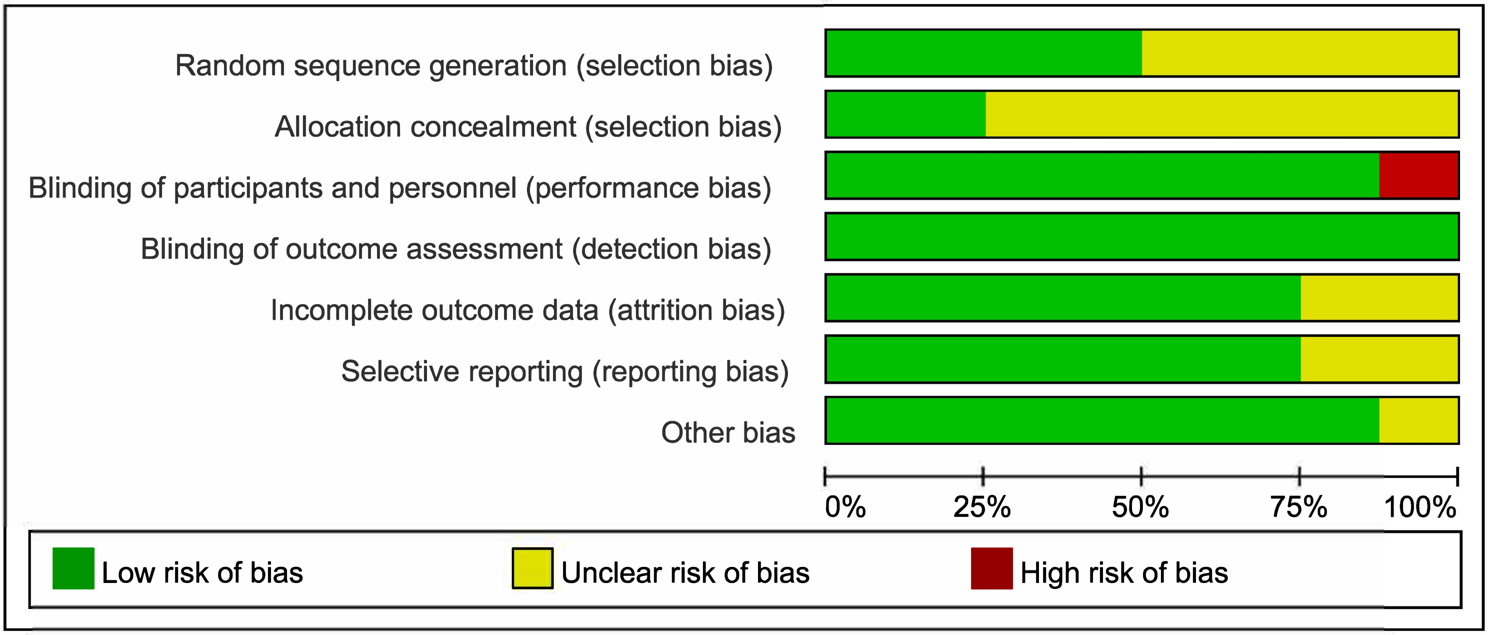

Supplementary Figure 2 Risk of bias summary
review authors' judgments about each risk of bias item for each included study
