# Supplementary material for: Comparative Efficacy and Safety of Targeted Therapies for Chronic Thromboembolic Pulmonary Hypertension: A Systematic Review and Network Meta-Analysis
Source: Can Respir J. 2021 Sep 1;2021:1626971. doi: 10.1155/2021/1626971 (PMC8426079; doi:10.1155/2021/1626971)
Supplement: Supplementary Materials — Supplementary Material 1. Details about data analysis. Supplementary Figure 1 A–E. Network plot for all outcomes. Supplementary Figure 2. Risk of bias summary. Supplementary Figure 3 A–E. Pairwise meta-analysis for 6MWD, BNP/NT-proBNP, NYHA/WHO FC improvement, PVR, and clinical worsening. Supplementary Table 1. Outcome measures are being used in each included RCT. [file 1626971.f1.zip › 1626971.f1/Supplementary Figure 3 A-E (1).pdf]

A

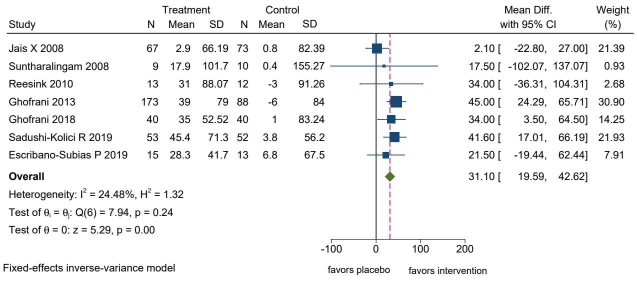

B

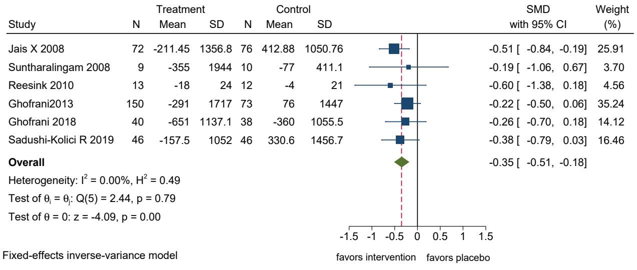

C

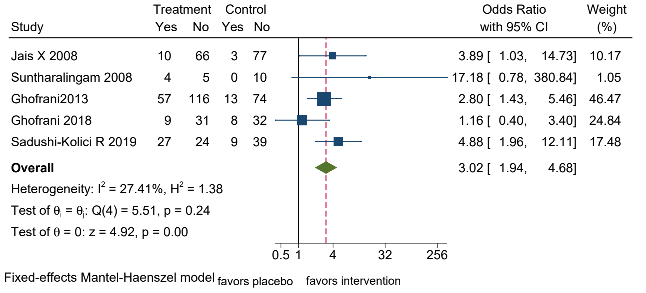

D

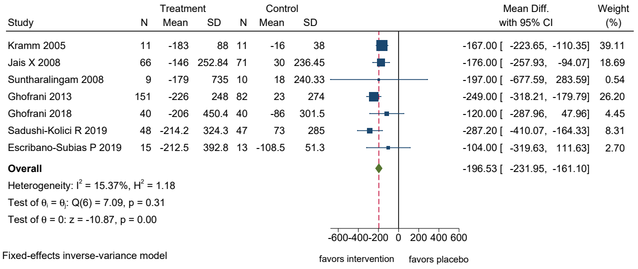

E

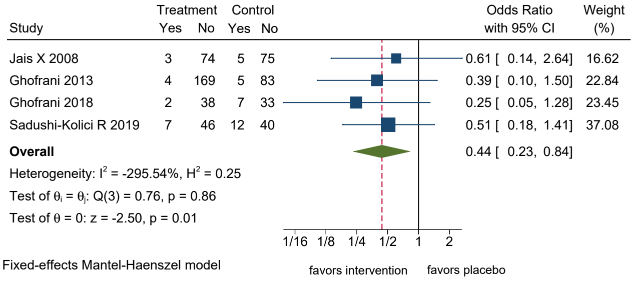

Supplementary Figure 3 A-E displays separately pairwise meta-analysis for 6MWD, BNP/NT-proBNP, NYHA/WHO FC improvement, PVR, and clinical worsening.
Data are shown for the fixed effects models. Error bars indicate 95％ CI. Rhombus represents the merging effect. $p<0.05$ for the overall estimate of the primary analysis is significant. Figure 3 A and Figure 3D were pooled by WMD. Figure 3 B was pooled by SMD. Figure 3 C and Figure 3 E were pooled by OR.
