# Supplementary material for: Comparative Efficacy and Safety of Targeted Therapies for Chronic Thromboembolic Pulmonary Hypertension: A Systematic Review and Network Meta-Analysis
Source: Can Respir J. 2021 Sep 1;2021:1626971. doi: 10.1155/2021/1626971 (PMC8426079; doi:10.1155/2021/1626971)
Supplement: Supplementary Materials — Supplementary Material 1. Details about data analysis. Supplementary Figure 1 A–E. Network plot for all outcomes. Supplementary Figure 2. Risk of bias summary. Supplementary Figure 3 A–E. Pairwise meta-analysis for 6MWD, BNP/NT-proBNP, NYHA/WHO FC improvement, PVR, and clinical worsening. Supplementary Table 1. Outcome measures are being used in each included RCT. [file 1626971.f1.zip › 1626971.f1/Supplementary Material 1 (1).docx]

Supplementary Material 1details about data analysis.

Among all five endpoints associated with the evaluation of efficacy and safety, three were continuous variables (6MWD, BNP/NT-proBNP, PVR), and two categorical variables (NYHA/WHO FC improvement, clinical worsening). All endpoints were extracted as change or improvement, that is, all outcomes were values in the terminal minus the baseline. During statistical analysis, the continuous variable was treated as weighted mean difference (WMD), except BNP/NT-proBNP treated as standardized mean difference (SMD) as a result of the measurement methods and units of each original research outcome varying, and the binary variables were calculated as odds ratio (OR). Meanwhile, their 2-tailed 95% confidence interval (CI) was also estimated to show significance. The interval containing 0 for WMD/SMD and 1 for OR predicted no significant difference. Statistical heterogeneity was tested using the I^2^ statistic (greater than 50% suggested substantial heterogeneity).

Stata software (version 16.0) was used to process the analysis.
